# Parasitological impact of 2-year preventive chemotherapy on schistosomiasis and soil-transmitted helminthiasis in Uganda

**DOI:** 10.1186/1741-7015-5-27

**Published:** 2007-09-03

**Authors:** Yaobi Zhang, Artemis Koukounari, Narcis Kabatereine, Fiona Fleming, Francis Kazibwe, Edridah Tukahebwa, J Russell Stothard, Joanne P Webster, Alan Fenwick

**Affiliations:** 1 Schistosomiasis Control Initiative, Department of Infectious Disease Epidemiology, Imperial College London, UK; 2 Vector Control Division, Ministry of Health, Kampala, Uganda; 3 Department of Zoology, Natural History Museum, London, UK

## Abstract

**Background:**

Schistosomiasis and soil-transmitted helminthiasis (STH) are among the neglected tropical diseases in Africa. A national control program for these diseases was initiated in Uganda during March 2003. Annual treatment with praziquantel and albendazole was given to schoolchildren in endemic areas and to adults in selected communities where local prevalence of *Schistosoma mansoni *in schoolchildren was high.

**Methods:**

The impact of the treatment program was monitored through cohorts of schoolchildren and adults. Their infection status with *S. mansoni *and STH was determined by parasitological examinations at baseline and at annual follow-ups. The prevalence and intensity of *S. mansoni *and STH before and after treatment were analyzed.

**Results:**

Two rounds of treatment significantly reduced the prevalence of *S. mansoni *infection in schoolchildren across three regions in the country from 33.4–49.3% to 9.7–29.6%, and intensity of infection from 105.7–386.8 eggs per gram of faeces (epg) to 11.6–84.1 epg. The prevalence of hookworm infection was reduced from 41.2–57.9% to 5.5–16.1%, and intensity of infection from 186.9–416.8 epg to 3.7–36.9 epg. The proportion of children with heavy *S. mansoni *infection was significantly reduced from 15% (95% CI 13.4–16.8%) to 2.3% (95% CI 1.6–3.0%). In adults, significant reduction in the prevalence and intensity of *S. mansoni *and hookworm infections was also observed. More importantly, the prevalence and intensity of both *S. mansoni *and hookworm infections in the cohorts of newly-recruited 6-year-olds who had never previously received treatment decreased significantly over 2 years: 34.9% (95% CI 31.9–37.8%) to 22.6% (95% CI 19.9–25.2%) and 171.1 epg (95% CI 141.5–200.7) to 72.0 epg (95% CI 50.9–93.1) for *S. mansoni*; and 48.4% (95% CI 45.4–51.5) to 15.9% (95% CI 13.6–18.2) and 232.7 epg (95% CI 188.4–276.9) to 51.4 epg (95% CI 33.4–69.5) for hookworms, suggesting a general decline in environmental transmission levels.

**Conclusion:**

Annual anthelminthic treatment delivered to schoolchildren and to adults at high risk in Uganda can significantly reduce the prevalence and intensity of infection for schistosomiasis and STH, and potentially also significantly reduce levels of environmental transmission of infection.

## Background

Schistosomiasis is a parasitic disease caused by infection with blood-fluke trematode schistosomes through contact with infective larvae in aquatic habitats of poor hygiene and sanitation that contain freshwater snails, the parasites' intermediate host. In Africa, two forms of human schistosomiasis occur: intestinal schistosomiasis due to *Schistosoma mansoni *and urinary schistosomiasis due to *Schistosoma haematobium*. By contrast, soil-transmitted helminthiasis (STH) is caused by infection with a group of intestinal nematode worms, most importantly in much of sub-Saharan Africa, the hookworms, *Ascaris lumbricoides *and *Trichuris trichiura*, through contact with parasite eggs or larvae that are present in the contaminated environment. Both schistosomiasis and STH are among the neglected tropical diseases that remain serious public health problems, posing unacceptable threats to human health and welfare, especially in the developing world. In total around 200 million people worldwide are estimated to be infected with schistosomes [1-3], and the numbers of STH infections are much greater [4-7]. The combined loss of 'disability-adjusted life years' due to the above parasitic infections is 43.5 million [6-8], only ranking behind those for lower respiratory infections (91.3 million), HIV/AIDS (84.5 million), diarrhoeal diseases (62 million), and malaria (46.5 million) [9,10]. Moreover, the disability-adjusted life years for schistosomiasis recently have been considered to greatly underestimate the true burden of the disease and are in need of reassessment [11-15]. Therefore, the actual total burden of these parasitic infections could be much greater. It is well documented that these infections have a debilitating effect on people's health, especially children – the most vulnerable group. Schistosomiasis alone could be responsible for 200000 deaths per year in sub-Saharan Africa, and STH could be responsible for 135000 deaths per year globally [14]. Despite these facts, the control of these neglected tropical diseases has not previously been given priority within the national health programs of many of the sub-Saharan African countries. The main reasons for this are, first, that many of these parasitic infections can be asymptomatic and less overtly life-threatening than diseases such as HIV/AIDS, tuberculosis, and malaria and, second, that the necessary anthelminthic drugs have been too expensive for widespread use within countries with limited financial resources.

In Uganda, intestinal schistosomiasis has long been known to be prevalent, being dominant in 38 out of 58 districts within regions surrounding Lakes Victoria and Albert and the Albertine Nile area [16-18]. While urinary schistosomiasis also occurs in Uganda, its distribution is confined to a few isolated foci within central Uganda [18]. Recent surveys conducted between 1998 and 2002 showed that 16.7 million people, out of a total population of 24 million people in 108 counties, were estimated to be at risk of infection with *S. mansoni *[18]. The level of infection in the human population was closely related to the proximity with local water bodies where daily water contact activities such as washing and bathing occur. Places with the highest prevalence were closer to the immediate shorelines of Lakes Victoria and Albert, and in certain locations infections were nearly universal (100%) [18]. Hookworm infections tend to be widespread throughout the country with a prevalence of up to 90%, while *A. lumbricoides *and *T. trichiura *infections tend to be more geographically restricted, being greatest in the southwestern districts and to a lesser extent in central and part of eastern Uganda (up to 89% and 68%, respectively) [19-22]. Although some individual treatment projects had been carried out in certain areas of the country, the implementation of the first systemic national control program for schistosomiasis and STH was not initiated until 2003, despite the fact that a very effective drug, praziquantel (PZQ), has been available for decades. The current program was made possible by the recent reduction in the price of drugs [23] and the financial and technical support from the Schistosomiasis Control Initiative, funded by the Bill and Melinda Gates Foundation.

By the beginning of 2005, two rounds of annual mass chemotherapy with PZQ and albendazole (ALB) had been delivered. Approximately 3.5 million schoolchildren and community adults at high risk had received treatment. Cohorts of schoolchildren and community adults were selected and followed-up annually to monitor the effect of the control program. Previous analysis showed that the treatment had significantly improved anaemia status, which was associated with schistosomiasis and hookworm infections, and clinical manifestations in schoolchildren [24,25]. The aim of the current paper is to assess in detail the significant impact of two rounds of mass chemotherapy in this control program upon the prevalence and intensities of these parasitic infections in different epidemiological settings, both on those treated individuals and more importantly, also on the new cohorts of 6-year-old untreated children, potentially reflective of the subsequent transmission risk in the environment.

## Methods

### National control program

**Figure 1:**
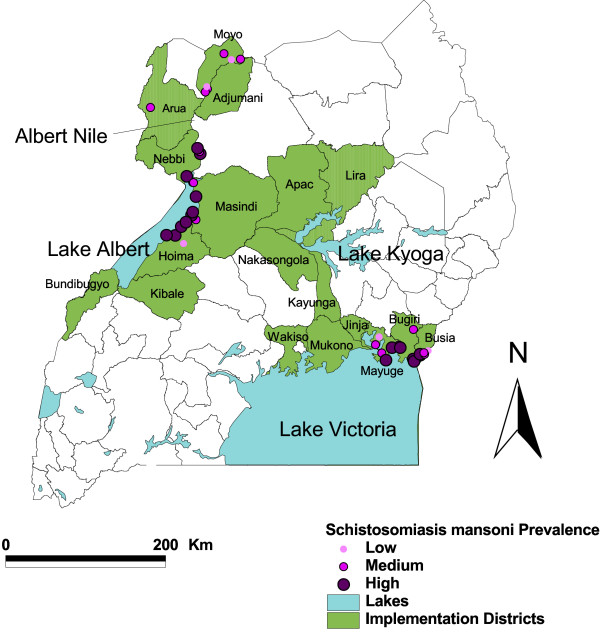
**Map of the initial program coverage areas and the cohort school location in Uganda**. The treatment program initially covered the 18 districts shown in green, and 37 schools shown as circles were selected from eight districts for follow-up monitoring.

The Ugandan national control program for schistosomiasis and STH was the first among the six in Africa supported by the Schistosomiasis Control Initiative. The control strategies used were school-based mass chemotherapy in children and selective chemotherapy in adults at high risk of schistosome infection, together with health education, following the World Health Organization guidelines [7]. The program started initially in 18 districts, which were known to have the highest prevalence of *S. mansoni *infections (Figure 1). The treatment strategy for each community was decided according to its endemic category using available epidemiological mapping data [18] and was based on the schistosomiasis prevalence in the areas: (1) where schistosomiasis prevalence was over 50% (high category), annual mass treatment was given to schoolchildren and community adults; (2) where schistosomiasis prevalence was 10–50% (medium category), annual mass treatment was given to schoolchildren only; and (3) where schistosomiasis prevalence was below 10% (low category), schoolchildren were treated in the first two rounds of treatment only [7,23]. The details of the control strategies and implementation of the program are described elsewhere [26]. The dosage of PZQ was 40 mg/kg body weight, co-administered with a single 400 mg ALB tablet. PZQ tablets were procured from Shin Poong (Seoul, Korea) and ALB from GlaxoSmithKline (Brentford, Middlesex, UK). The treatment received ethical clearance from the National Health System Local Research Ethics Committee of St Mary's Hospital, London as well as approval from the Ministry of Health and the National Council of Science and Technology, Kampala, Uganda.

### Monitoring cohorts and parasitological diagnosis

**Figure 2:**
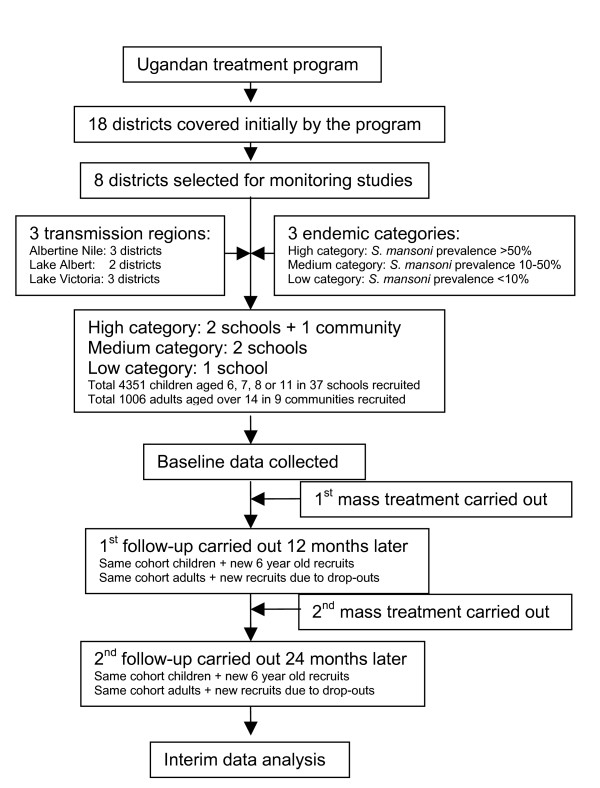
Diagram of the study structure.

In order to monitor the impact of the treatment, identifiable cohorts of schoolchildren and community adults were recruited and examined at baseline and at follow-ups (Figure 2). The details of cohort selection and sample sizes were described previously [24,27]. In brief, 37 primary schools in eight districts were selected for monitoring studies, representing three different transmission regions, the Albertine Nile region (Nebbi, Moyo, and Arua districts), the Lake Albert region (Hoima and Masindi districts), and the Lake Victoria region (Busia, Bugiri, and Mayuge districts), and three endemic categories as described above. Within each district, five schools were selected according to endemic categories: two from the high category, two from the medium category, and one from the low category, except that only one school was surveyed in Arua, and six in Moyo due to logistical reasons. Within each school, 120 children were selected randomly on the basis of age (6-, 7-, 8-, or 11-year-olds) to include approximately 15 boys and 15 girls in each age group. We also selected nine communities from eight districts where the initial prevalence in the school was high (>50%). Approximately 120 people over the age of 14 were recruited from each community on a voluntary basis. A total of 4351 children and 1006 adults were recruited and examined at baseline. The same cohorts of children and adults, if traced, were re-examined 12 and 24 months later, each in the same month of the year as when the baseline survey was performed and before the next round of treatment taking place. At each follow-up an additional 15 boys and 15 girls of 6 years of age were recruited from each school. Due to the high dropout rate in the adult cohorts, additional voluntary adults were examined in each community at each follow-up. On each occasion, faecal samples from all individuals were examined using the single modified Kato-Katz thick smear [28] for schistosomiasis and STH. The focal egg counts for each of the four parasites were recorded, and the prevalence and arithmetic mean intensity of infection (epg) including both positive and negative individuals was calculated [29]. To minimize the measurement bias on the parasitological data, all Kato-Katz slides were read within 2 h of preparation to avoid the degeneration of hookworm eggs, and 10% of the total slides were randomly selected and read by a second experienced microscopist for quality control. Prior to each examination, written informed consents were obtained from each participant for adults and from head teachers for school children with verbal consents from parents.

### Data management and analysis

Each individual in the cohorts was given a specific, but anonymous, identification number. Once collected, all data were computerized into the database according to the identification numbers. Of 4351 schoolchildren recruited at baseline, 1704 were successfully examined at baseline and re-examined at both follow-ups, and therefore, have the three complete sets of longitudinal parasitological data. These longitudinal data, together with three sets of cross-sectional data from the 6-year-old children, were presented in the current descriptive analysis for schoolchildren. The baseline characteristics of the children successfully followed up were similar to those dropped-out in mean age (8.0 vs 8.1), sex ratio (1.04 vs 1.02), hookworm prevalence (51% vs 52.2%), and hookworm intensity of infection (302.9 epg vs 283.8 epg) (p > 0.05), while *S. mansoni *prevalence and intensity of infection were higher in those dropped out (47.5% and 314.1 epg) than in those followed up (42.4% and 219.6 epg) (p < 0.05). For adult data, as the majority of original adult recruits could not be followed-up for the study period (very few have the three complete sets of longitudinal data), the cross-sectional data from all adults examined at each visit were presented here. Frequency tables together with 95% confidence intervals (CIs) were obtained using software SAS v.8 (SAS Institute, Cary, NC, USA).

## Results

### Impact on the prevalence and intensity of intestinal schistosomiasis in schoolchildren

The results are summarized in Table 1. The overall prevalence was 42.4% at baseline. In different regions the Lake regions had a higher prevalence than the Albertine Nile region (Table 1). The average prevalence was 77.2% in high category schools, 29.9% in medium category schools, and 2.1% in low category schools. The prevalence of schistosome infection was not significantly different between boys and girls (Table 1). After PZQ treatment, the overall prevalence was significantly reduced to 26.8% after the first round of treatment and to 17.9% after the second round of treatment, a total reduction of 57.8%. In different regions, a greater reduction (71%) was shown in the Nile region than in the Lake Victoria region (58.2%) or in the Lake Albert region (40%). In different endemic categories, the highest percentage reduction in prevalence (73.2%) was shown in the medium category schools, while the prevalence in high or low category schools was reduced by half. A similar degree of reduction in schistosome prevalence was observed in both genders after treatments, as shown in Table 1.

**Table 1:** *S. mansoni *prevalence and intensity of infection in the school children before and after treatment

	**Number of subjects**	**Baseline**	**Follow-up Year 1**	**Follow-up Year 2**	**Overall reduction**
**Prevalence**					
Overall prevalence (%)	1 704	42.4 (40.0–44.7)	26.8 (24.7–28.9)	17.9 (16.1–19.7)	57.8%
Regions					
Albertine Nile	638	33.4 (29.7–37.0)	18.0 (15.0–21.0)	9.7 (7.4–12.0)	71.0%
Lake Albert	335	49.3 (43.9–54.6)	39.1 (33.9–44.3)	29.6 (24.7–34.4)	40.0%
Lake Victoria	731	47.1 (43.4–50.7)	28.7 (25.4–32.0)	19.7 (16.8–22.6)	58.0%
Original category					
High	620	77.2 (73.9–80.5)	56.9 (53.0–60.8)	38.5 (34.6–42.3)	50.1%
Medium	795	29.9 (26.7–33.1)	12.8 (10.5–15.1)	8.0 (6.2–9.9)	73.2%
Low	289	2.1 (0.4–3.7)	0.7 (0.0–1.7)	1.0 (0.0–2.2)	52.4%
Sex					
Boys	879	41.5 (38.3–44.8)	27.1 (24.1–30.0)	18.3 (15.8–20.9)	55.9%
Girls	821	43.2 (39.9–46.6)	26.4 (23.4–29.5)	17.3 (14.7–19.9)	60.0%
**Intensity of infection**					
Overall					
Mean epg	1 704	219.6 (191.8–247.4)	73.4 (58.7–88.0)	37.4 (27.4–47.5)	83.0%
0 epg (%)	-	57.6 (55.3–60.0)	73.2 (71.1–75.3)	82.1 (80.3–83.9)	-
1–99 epg (%)	-	16.2 (14.4–17.9)	14.6 (12.9–16.2)	11.5 (10.0–13.0)	-
100–399 epg (%)	-	11.1 (9.6–12.6)	7.3 (6.1–8.6)	4.1 (3.2–5.1)	-
≥ 400 epg (%)	-	15.1 (13.4–16.8)	4.9 (3.8–5.9)	2.3 (1.6–3.0)	-
Region					
Albertine Nile	638	105.7 (80.6–130.8)	44.2 (30.7–57.5)	11.6 (6.1–17.1)	89.0%
Lake Albert	335	386.8 (300.3–473.3)	165.9 (105.0–226.9)	84.1 (47.7–120.6)	78.3%
Lake Victoria	731	242.3 (197.2–287.4)	56.4 (41.0–71.8)	38.6 (23.2–54.0)	84.1%
Original category					
High	620	522.3 (454.6–590.0)	175.8 (138.6–213.0)	86.6 (63.8–109.3)	83.4%
Medium	795	60.2 (45.5–74.8)	20.2 (10.5–29.8)	12.7 (1.2–24.2)	78.9%
Low	289	10.1 (0.0–12.7)	0.3 (0.0–1.0)	0.4 (0.0–1.0)	96.0%
Sex					
Boys	879	233.1 (190.8–275.5)	77.8 (53.4–102.1)	32.8 (23.2–42.4)	85.9%
Girls	821	205.9 (170.2–241.6)	68.2 (52.4–84.0)	42.5 (24.4–60.6)	79.4%

Figures in () indicate 95% confidence interval.

At baseline, 15% of the children had heavy *S. mansoni *infections (≥ 400 epg), and the arithmetic mean intensity of infection of all participants was 219.6 epg (Table 1). There was no significant difference in the mean intensities between the boys and the girls. In different regions, the highest mean intensity of schistosome infection (386.8 epg) was found in the Lake Albert region while the lowest mean intensity (105.7 epg) was recorded in the Albertine Nile region. In schools of different endemic categories, the heaviest infection was in schools of high endemic category with an arithmetic mean intensity of 522.3 epg. After two rounds of PZQ treatment, the proportion of the heavy infections with *S. mansoni *was reduced to only 2.3% at the second follow-up (Table 1). The overall arithmetic mean intensity of infection was reduced by 83%. Similar reduction was observed in both boys and girls after treatment, with a total reduction of 85.9% and 79.4% respectively at the second follow-up. Although the percentage reduction in mean intensity looks higher in the Albertine Nile region, the absolute reduction inepg in both lake regions was much steeper: 300 epg (Lake Albert) and 200 epg (Lake Victoria), respectively. Similarly in different endemic categories, the most noticeable reduction inepg was in schools of high category, by over 400 epg.

### Impact on the prevalence and intensity of intestinal schistosomiasis in community adults

The results for adults are from the cross-sectional data (Table 2). Overall prevalence of *S. mansoni *infection was 57.4% at baseline, higher in males than in females. As in children, the prevalence of *S. mansoni *infection in adults in the Lake regions was higher than in the Albertine Nile region (Table 2). Two rounds of treatment achieved a total reduction of 82.9% in the overall prevalence. There was no difference in prevalence between males and females after two rounds of treatment. The prevalence in both genders was significantly reduced from baseline by 85.6% in males and 79.5% in females. As in Table 2, the percentage reduction in three regions was 72.9% in the Lake Albert region, 82.5% in the Lake Victoria region, and 85.8% in the Albertine Nile region.

**Table 2:** *S. mansoni *prevalence and intensity of infection in the community adults before and after treatment (cross-sectional data)

	**Baseline**	**Follow-up Year 1**	**Follow-up Year 2**	**Overall reduction**
**Prevalence**				
Overall prevalence (%)	57.4 (54.1–60.6) (n = 903)	34.7 (31.1–38.2) (n = 689)	9.8 (6.9–12.8) (n = 397)	82.9%
Regions				
Albertine Nile	43.1 (35.9–50.3) (n = 181)	16.1 (10.2–22.0) (n = 149)	6.1 (1.7–10.6) (n = 114)	85.8%
Lake Albert	65.6 (60.2–70.9) (n = 302)	42.9 (35.2–50.7) (n = 156)	17.8 (6.6–28.9) (n = 45)	72.9%
Lake Victoria	57.6 (52.9–62.4) (n = 420)	38.5 (33.7–43.4) (n = 384)	10.1 (6.3–13.9) (n = 238)	82.5%
Sex				
Male	64.6 (60.2–69.1) (n = 441)	40.1 (34.5–45.7) (n = 294)	9.3 (5.3–13.2) (n = 205)	85.6%
Female	48.2 (43.4–53.0) (n = 413)	28.5 (23.3–33.7) (n = 288)	9.9 (5.7–14.2) (n = 191)	79.5%
**Intensity of infection**				
Overall				
Mean epg	251.6 (213.7–289.6) (n = 903)	80.4 (61.1–99.7) (n = 689)	19.6 (4.9–34.3) (n = 397)	92.2%
0 epg (%)	42.6 (39.4–45.9) (n = 385)	65.3 (61.7–68.9) (n = 450)	90.2 (87.3–93.1) (n = 358)	-
1–99 epg (%)	23.7 (20.9–26.5) (n = 214)	19.3 (16.4–22.3) (n = 133)	6.8 (4.3–9.3) (n = 27)	-
100–399 epg (%)	16.8 (14.4–19.3) (n = 152)	10.2 (7.9–12.4) (n = 70)	2.0 (0.6–3.4) (n = 8)	-
≥ 400 epg (%)	16.8 (14.4–19.3) (n = 152)	5.2 (3.6–6.9) (n = 36)	1.0 (0.0–2.0) (n = 4)	-
Region				
Albertine Nile	67.6 (46.3–89.0) (n = 181)	31.0 (9.4–52.5) (n = 149)	2.5 (0.1–4.8) (n = 116)	96.3%
Lake Albert	411.2 (321.6–500.7) (n = 302)	131.9 (74.9–189.0) (n = 156)	62.0 (0.0–167.6) (n = 48)	84.9%
Lake Victoria	216.2 (169.7–262.6) (n = 420)	78.7 (54.5–102.9) (n = 384)	18.4 (5.9–30.8) (n = 247)	91.5%
Sex				
Male	346.9 (285.0–408.8) (n = 441)	101.8 (65.8–137.9) (n = 294)	19.6 (0.0–44.4) (n = 205)	94.3%
Female	154.9 (107.6–202.2) (n = 413)	69.2 (42.9–95.6) (n = 288)	19.7 (4.6–34.9) (n = 191)	87.3%

Figures in () indicate 95% confidence interval; n, number of subjects in the group.

In these adults, 16.8% of population had heavy *S. mansoni *infections, and the arithmetic mean intensity of infection was 251.6 epg at baseline. Males were significantly more heavily infected than females. Generally speaking, those in the Lake Albert region were most heavily infected, and those in the Albertine Nile region were least heavily infected. As shown in Table 2, after two rounds of treatment the arithmetic mean intensity of *S. mansoni *infection was dramatically reduced to 19.6 epg, a reduction of 92.2%. The proportion of heavy infections decreased to only 1%. There was no difference in intensity of infection between males and females after two rounds of treatment, with a greater degree of reduction shown in males.

### Impact on the prevalence and intensity of STH in schoolchildren

The overall prevalence of hookworm infection was 50.9% at baseline. It was slightly higher in the Lake Victoria region than in the Albertine Nile region or in the Lake Albert region, respectively (Table 3). There was no significant difference in prevalence of hookworm infection between boys and girls at baseline. After ALB treatment, the overall prevalence was reduced to 24.1% after the first treatment and to 10.7% after the second treatment, respectively, representing a reduction of 79%. Substantive decreases in prevalence were seen in all three regions, and even after one treatment the hookworm infection in the Lake Albert and the Nile regions was reduced by over 75%. Both boys and girls showed similar improvement in reducing prevalence of hookworm infections.

**Table 3:** Soil-transmitted helminth prevalence and intensity of infection in the school children before and after treatment

	**No of subjects**	**Baseline**	**Follow-up Year 1**	**Follow-up Year 2**	**Overall reduction**
**Hookworm**					
**Prevalence (%)**					
Overall	1 704	50.9 (48.6–53.3)	24.1 (22.1–26.2)	10.7 (9.3–12.2)	79.0%
Regions					
Albertine Nile	637	48.0 (44.2–51.9)	11.6 (9.1–14.1)	5.5 (3.7–7.3)	88.5%
Lake Albert	335	41.2 (35.9–46.5)	8.4 (5.4–11.3)	9.0 (5.9–12.0)	78.2%
Lake Victoria	732	57.9 (54.3–61.5)	42.2 (38.6–45.8)	16.1 (13.5–18.8)	72.2%
Sex					
Boys	877	53.5 (50.2–56.8)	27.3 (24.3–30.2)	12.2 (10.0–14.4)	77.2%
Girls	823	48.2 (44.8–51.7)	20.8 (18.0–23.6)	9.2 (7.3–11.2)	80.9%
**Intensity of infection (epg)**					
Overall					
Mean epg	1 704	309.4 (232.4–386.4)	76.8 (62.9–90.7)	21.9 (13.8–30.1)	92.9%
0 epg (%)	-	49.0 (46.7–51.4)	75.9 (73.8–77.9)	89.3 (87.8–90.7)	-
1–1 999 epg (%)	-	48.4 (46.0–50.7)	23.5 (21.5–25.5)	10.6 (9.2–12.1)	-
2 000–3 999 epg (%)	-	1.6 (1.0–2.2)	0.5 (0.1–0.8)	0.1 (0.0–0.2)	-
≥ 4 000 epg (%)	-	1.0 (0.5–1.5)	0.1 (0.0–0.3)	0.1 (0.0–0.2)	-
Region					
Albertine Nile	637	186.9 (148.0–225.8)	29.6 (17.1–42.2)	3.7 (1.7–5.7)	98.0%
Lake Albert	335	307.4 (1.2–613.6)	22.8 (9.3–36.2)	23.9 (0.0–51.4)	92.2%
Lake Victoria	732	416.8 (310.0–523.5)	142.6 (113.4–171.8)	36.9 (22.8–50.9)	91.1%
Sex					
Boys	877	370.3 (229.5–511.1)	76.7 (60.5–92.8)	19.4 (12.3–26.5)	94.8%
Girls	823	244.5 (190.7–298.4)	77.3 (54.3–100.4)	24.7 (9.6–39.8)	89.9%
***Ascaris lumbricoides***					
Overall prevalence (%)	1 700	2.8 (2.0–3.6)	1.6 (1.0–2.3)	0.6 (0.3–1.0)	-
Intensity of infection (epg)	1 700	95.4 (26.4–164.3)	20.0 (4.9–35.1)	16.2 (3.5–28.9)	83.0%
***Trichuris trichiura***					
Overall prevalence (%)	1 700	2.2 (1.5–2.9)	2.5 (1.7–3.2)	1.6 (1.0–2.2)	-
Intensity of infection (epg)	1 700	3.3 (0.9–5.6)	1.8 (1.0–2.7)	3.05 (0.0–7.15)	-

Figures in () indicate 95% confidence interval.

Most of the hookworm infections were light infections at baseline (<2000 epg) as in Table 3 with an estimated arithmetic mean intensity of infection of 309.4 epg. There was no significant difference in mean intensity between genders, and the highest mean intensity was found around Lake Victoria (Table 3). After two rounds of treatment with ALB, the arithmetic mean intensity of infection significantly decreased to 21.9 epg. The percentage reduction in the mean intensity was similar between the genders and in the different regions; however, the absolute reduction in the mean intensity was much greater in the Lake Albert and Lake Victoria regions, where the initial mean intensity was higher.

The prevalence of *A. lumbricoides *or *T. trichiura *was relatively low in the cohort areas (Table 3). The prevalence of *A. lumbricoides *was reduced from 2.8% to 0.6%, and the intensity of infection was reduced by 83%, however the impact was negligible because of the very light infections in these areas. The prevalence of *T. trichiura *did not show much change, maintaining at around a 2% level, and there was no significant change in the mean intensity of *T. trichiura *infections either. Analysis of *A. lumbricoides *or *T. trichiura *infection by different epidemiological settings did not show any statistical differences, therefore, such details are not presented in this paper.

### Impact on the prevalence and intensity of STH in community adults

At baseline, hookworm prevalence was 31.7%, and there was no significant difference between males and females, as shown in Table 4. In contrast to schistosome infection, the prevalence of hookworm infection was significantly lower in the Lake Albert region than in the Lake Victoria region and in the Albertine Nile region. After two rounds of treatment, the prevalence decreased by 61.8% in total, 68.2% in males, and 56.1% in females. Significant reduction was shown in all three regions (Table 4).

**Table 4:** Soil-transmitted helminth prevalence and intensity of infection in the community adults before and after treatment

	**Baseline**	**Follow-up Year 1**	**Follow-up Year 2**	**Overall reduction**
**Hookworm**				
**Prevalence (%)**				
Overall	31.7 (28.6–34.7) (n = 903)	36.1 (32.6–39.7) (n = 689)	12.1 (8.9–15.3) (n = 397)	61.8%
Regions				
Albertine Nile	34.3 (27.3–41.2) (n = 181)	28.2 (21.0–35.4) (n = 149)	7.0 (2.3–11.7) (n = 114)	79.6%
Lake Albert	14.2 (10.3–18.2) (n = 302)	12.2 (7.1–17.3) (n = 156)	0.0 (NA) (n = 45)	100%
Lake Victoria	43.1 (38.4–47.8) (n = 420)	49.0 (44.0–54.0) (n = 384)	16.8 (12.1–21.6) (n = 238)	61.0%
Sex				
Male	27.7 (23.5–31.8) (n = 441)	30.3 (25.0–35.5) (n = 294)	8.8 (4.9–12.7) (n = 205)	68.2%
Female	35.8 (31.2–40.5) (n = 413)	36.1 (30.6–41.7) (n = 288)	15.7 (10.6–20.9) (n = 191)	56.1%
**Intensity of infection**				
Overall				
Mean epg	121.9 (92.6–151.1) (n = 903)	142.6 (101.5–183.7) (n = 689)	34.3 (15.5–53.1) (n = 397)	71.9%
0 epg (%)	68.3 (65.3–71.4) (n = 617)	63.9 (60.3–67.4) (n = 440)	87.9 (84.7–91.1) (n = 349)	-
1–1 999 epg (%)	30.7 (27.7–33.7) (n = 277)	35.0 (31.4–38.5) (n = 241)	11.8 (8.7–15.0) (n = 47)	-
2 000–3 999 epg (%)	0.7 (0.1–1.2) (n = 6)	0.4 (0.0–0.9) (n = 3)	0.3 (NA) (n = 1)	-
≥ 4 000 epg (%)	0.3 (0.0–0.7) (n = 3)	0.7 (0.1–1.4) (n = 5)	0.00 (NA) (n = 0)	-
Regions				
Albertine Nile	79.3 (49.4–109.2) (n = 181)	54.9 (18.1–91.7) (n = 149)	10.1 (0.0–20.5) (n = 114)	87.3%
Lake Albert	47.3 (10.8–83.7) (n = 302)	38.2 (10.8–65.6) (n = 156)	0.00 (NA) (n = 45)	100%
Lake Victoria	193.8 (138.7–248.9) (n = 420)	219.0 (148.3–289.7) (n = 384)	52.3 (21.5–83.1) (n = 238)	73.0%
Sex				
Male	99.4 (63.2–135.6) (n = 441)	137.6 (59.8–215.3) (n = 294)	18.6 (4.8–32.5) (n = 205)	81.3%
Female	154.1 (103.3–205.0) (n = 413)	124.1 (88.9–159.2) (n = 288)	51.3 (15.1–87.4) (n = 191)	66.7%
***Ascaris lumbricoides***				
Overall prevalence (%)	1.0 (0.4–1.7) (n = 903)	1.5 (0.6–2.3) (n = 689)	0.5 (0.0–1.2) (n = 397)	-
Mean epg	2.4 (0.0–5.6) (n = 903)	6.3 (0.6–11.9) (n = 689)	3.6 (0.0–10.5) (n = 397)	-
***Trichuris trichiura***				
Overall prevalence (%)	1.3 (0.6–2.1) (n = 903)	1.2 (0.4–2.0) (n = 689)	0.8 (0.0–1.6) (n = 397)	-
Mean epg	0.6 (0.2–1.1) (n = 903)	1.6 (0.0–3.4) (n = 689)	0.7 (0.0–1.7) (n = 397)	-

Figures in () indicate 95% confidence interval; n, number of subjects in the group; NA, not available.

Hookworm infections in adults were mainly light infections (Table 4). Arithmetic mean intensity of infection was 121.9 epg at baseline, and relatively heaviest infections were seen in the Lake Victoria region among three different regions. After two rounds of treatment, the intensity of infection decreased by a near 72% reduction in total. A significant reduction was observed in all three regions (Table 4). *A. lumbricoides *and *T. trichiura *infections were observed at a very low level (around 1%), and no significant impact was seen on these by the treatment.

### Impact on potential transmission: prevalence and intensity of infection in new 6-year-old untreated cohorts

Cohorts of the 6-year-olds from each school were recruited at baseline and at each of both follow-ups. These new cohort children were the first year pupils who had not had the history of previous treatment before they joined the school. Any children indicating, during the questionnaire survey, that they might have received treatment were excluded from analysis here. The data from these children should provide an indicator for the transmission levels in the areas. As shown in Table 5, at baseline, 34.9% of the 6-year-old children were infected with *S. mansoni*, and 48.4% infected with hookworms. After two rounds of treatment the prevalence and intensity of both *S. mansoni *and hookworm infections in the untreated 6-year olds significantly decreased (Table 5). A greater reduction was shown in hookworm infections. Even after one treatment a significant decrease was seen in both hookworm prevalence and intensity of infection. Such reduction was prominent in all three different regions, with the highest reduction in both *S. mansoni *and hookworm infections in the Albertine Nile region (Table 5).

**Table 5:** Prevalence and intensity of *S. mansoni *and hookworm infections in the newly-recruited cohorts of previously untreated 6-year-old children before and after treatment

	Baseline	Follow-up Year 1	Follow-up Year 2	Overall reduction
***S. mansoni***				
**Prevalence (%)**				
Overall	34.9 (31.9–37.8) (n = 1 018)	27.7 (25.0–30.4) (n = 1 075)	22.6 (19.9–25.2) (n = 961)	35.2%
Region				
Albertine Nile	22.4 (17.9–26.9) (n = 330)	12.9 (9.5–16.4) (n = 371)	11.3 (7.9–14.6) (n = 346)	49.6%
Lake Albert	47.5 (41.3–53.6) (n = 255)	42.4 (36.7–48.1) (n = 288)	32.3 (26.3–38.4) (n = 229)	32.0%
Lake Victoria	37.0 (32.4–41.5) (n = 433)	30.8 (26.3–35.2) (n = 416)	26.9 (22.5–31.4) (n = 386)	27.3%
**Intensity of infection (epg)**				
Overall	171.1 (141.5–200.7) (n = 1 018)	119.9 (92.1–147.8) (n = 1 075)	72.0 (50.9–93.1) (n = 961)	57.9%
Region				
Albertine Nile	93.7 (53.4–133.9) (n = 330)	42.7 (18.9–66.5) (n = 371)	10.8 (6.1–15.4) (n = 346)	88.5%
Lake Albert	152.9 (111.9–193.8) (n = 433)	84.2 (55.9–112.5) (n = 416)	86.2 (59.2–113.3) (n = 386)	43.6%
Lake Victoria	302.3 (223.9–380.7) (n = 255)	271.1 (182.7–359.5) (n = 288)	140.6 (65.6–215.6) (n = 229)	53.5%
**Hookworms**				
**Prevalence (%)**				
Overall	48.4 (45.4–51.5) (n = 1 018)	24.7 (22.1–27.3) (n = 1 075)	15.9 (13.6–18.2) (n = 961)	67.1%
Region				
Albertine Nile	46.1 (40.7–51.4) (n = 330)	14.0 (10.5–17.6) (n = 371)	10.7 (7.4–14.0) (n = 346)	76.8%
Lake Albert	39.6 (33.6–45.6) (n = 255)	10.8 (7.2–14.4) (n = 287)	12.2 (8.0–16.5) (n = 229)	69.2%
Lake Victoria	55.4 (50.8–60.2) (n = 433)	43.6 (38.9–48.4) (n = 417)	22.8 (18.6–27.0) (n = 386)	58.8%
**Intensity of infection (epg)**				
Overall	232.7 (188.4–276.9) (n = 1 018)	84.6 (63.6–105.6) (n = 1 075)	51.4 (33.4–69.5) (n = 961)	77.9%
Region				
Albertine Nile	184.1 (137.2–231.0) (n = 330)	39.7 (6.7–72.7) (n = 371)	27.8 (8.4–47.3) (n = 346)	84.9%
Lake Albert	171.0 (97.4–244.7) (n = 255)	20.2 (9.6–30.8) (n = 288)	32.2 (4.5–59.8) (n = 229)	81.2%
Lake Victoria	306.0 (218.6–393.4) (n = 433)	168.9 (124.9–212.8) (n = 416)	84.0 (46.0–122.0) (n = 386)	72.5%

Figures in () indicate 95% confidence intervals; n, number of subjects in the group.

## Discussion

Baseline data from 37 primary schools and nine communities in eight districts illustrated the significant burden of schistosomiasis and STH in Uganda. Prevalence of *S. mansoni *infection was nearly 100% in many schools, and 15% of children were heavily infected (≥ 400 epg). Hookworm infection was widespread in all areas, with an average prevalence of 50% in children. It was evident that the burden of these parasitic infections had not changed in the country until the current Schistosomiasis Control Initiative – supported national control program initiated in 2003 [16,18,21,30,31]. The main control strategies of the current program include chemotherapy and health education, particularly targeting schoolchildren. According to the World Health Assembly resolution 54.19, at least 75% of all school-aged children at risk of morbidity due to schistosomiasis and STH should be given regular treatment by 2010. The treatment coverage of school-aged children in our program has been shown to be well above the 75% threshold [26]. Our data suggest that this has resulted in a remarkable reduction of the burden due to these parasitic infections [24,25,27]. After two rounds of treatment with PZQ and ALB, significant reduction was observed in both prevalence and, more importantly, intensity of infection of *S. mansoni *and hookworms.

Two rounds of treatment in adults also showed a tremendous impact on the prevalence and intensity of *S. mansoni *and hookworm infections. However, it was noticed that the prevalence and intensity of hookworm infections were not reduced after the first round of treatment. This might have been due to the high dropout rate of adults from baseline and the recruitment of additional (different) individuals at follow-ups. It should also be noted that our adult data were obtained from the communities where the original *S. mansoni *prevalence in schoolchildren was high (>50%). Therefore, the adult data represent the situation only in such areas.

To evaluate the potential longer-term impact of the program, we examined the infections in the 6-year-old children who had not received treatment previously as they had only recently started attending school. It might be an important observation that both *S. mansoni *and hookworm infections in this group of children significantly decreased after two rounds of treatment (Table 5), suggesting that the level of environmental transmission in these areas could have been reduced because of overall reductions in excreted eggs from infected children, or owing to altered contaminating behaviours of the children as health education activities concurrent with administration of anthelmintics took place. The highest reduction in both *S. mansoni *and hookworm infections in the Albertine Nile region suggests a relatively lower transmission of the diseases in the region. For schistosomiasis a simple explanation is available: the Albertine Nile offers less convenient platforms for human water contact than the shorelines of the lakes owing to the increased riverine current and dangerously steep shelving banks.

The ultimate goal of the current control strategy recommended by the World Health Organization is to reduce the morbidity due to schistosomiasis and STH. The consequence of these parasitic infections has been well studied and documented. Apart from the liver and spleen pathology caused by intestinal schistosomiasis, anaemia, growth stunting, and cognitive impairment are also linked to these infections [14,32,33]. Our data also showed a strong link between anaemia and both *S. mansoni *and hookworm infections [24]. By studying two neighbouring communities in Uganda, Booth and colleagues showed that the development of hepatosplenic morbidity due to *S. mansoni *can be critically related to the duration of exposure to *S. mansoni *infections [34]. Therefore, repeated treatment as early in childhood as possible is particularly important to avoid the devastating consequences at a later stage of life [35]. And, indeed, one round of treatment has already shown a sign of improvement in children's health [24]. Distended abdomen syndrome in schoolchildren, as defined by an abdominal circumference ratio, was shown to likely improve after the first round of treatment [27]. With the program progressing, and more treatment and health education being delivered, long-lasting health benefits will undoubtedly be brought to Ugandan children.

In line with the previous data [21], infections with *A. lumbricoides *and *T. trichiura *in the current study areas are relatively low, and they appear to be more focally distributed in certain parts of the country and do not constitute a major health problem in our study areas. It is noticed from the present data that ALB efficacy at the current dose level (400 mg per person) was high on hookworms and on *A. lumbricoides *in children, as the prevalence and intensity of both infections decreased significantly after treatment. However, in agreement with previous reports by others [36-38], this dose was ineffective for the treatment of *T. trichiura*. As the program progresses and treatment is extended to southwestern Uganda, where *T. trichiura *infection rates could be as high as 100% (NK, unpublished data), a different treatment regimen, e.g., multiple treatment with ALB, should be considered to achieve better cure rates on *T. trichiura *[5,39].

Despite the progress made, it was generally observed that in cohort schools close to lakeshores, the prevalence and intensity of *S. mansoni *infection remained relatively high even after two rounds of treatment (data not shown). This could imply that transmission at these locations is persistently at a high level, and special attention should be devoted to such locations to bring about a further decline in transmission. However, a high treatment coverage rate is instrumental in controlling schistosomiasis transmission [40]. Although a minimum 80% coverage in the schools has been achieved at any time point in this program, frequent migration of people particularly from the fishing communities, some of whom are from neighbouring countries, and absence from school of some school-aged children remain significant hurdles to the successful implementation of the control program, particularly in terms of ensuring repeated treatment. A concerted action of all neighbouring countries to offer treatment in the neighbouring states would significantly improve the situation. The current implementation of the control program is being integrated with the national child health days in Uganda, and this might also help to improve the treatment coverage in school-aged children. An epidemiological remapping of the current endemic status of these parasitic infections amid the ongoing control program is now being carried out. The results will enable Uganda to identify those problem areas where transmission is persistently high so as to adjust the treatment strategy in each community to further bring down the level of infections, hence morbidity. Efforts are also being made to find additional funds to sustain the impact achieved in the long term.

## Conclusion

The current study has clearly demonstrated the significant impact of a national control program in sub-Saharan Africa at reducing the prevalence and intensity of schistosomiasis and STH and even potential environmental transmission of these diseases. We therefore set an example of successful implementation of a national control program to combat these important neglected tropical diseases in underdeveloped countries.

## Competing interests

The author(s) declare that they have no competing interests.

## Authors' contributions

AF obtained funding and was the principal investigator. JPW, JRS, and AF participated in the design of data collection. YZ, NK, FF, FK, ET, and JRS participated in data collection. YZ drafted and revised the manuscript. AK carried out statistical analysis. All authors contributed to the revision of the manuscript and agreed on submission.

## Pre-publication history

The pre-publication history for this paper can be accessed here:


